# Repositioning Clofazimine as a Macrophage-Targeting Photoacoustic Contrast Agent

**DOI:** 10.1038/srep23528

**Published:** 2016-03-22

**Authors:** Rahul K. Keswani, Chao Tian, Tyler Peryea, Gandikota Girish, Xueding Wang, Gus R. Rosania

**Affiliations:** 1 Department of Pharmaceutical Sciences, College of Pharmacy, University of Michigan, Ann Arbor, MI 48109; 2 Department of Radiology, University of Michigan, Ann Arbor, MI 48109; 3 Department of Biomedical Engineering, University of Michigan, Ann Arbor, MI 48109; 4 National Center for Advancing Translational Sciences (NCATS), National Institutes of Health, Rockville, MD 20850.

## Abstract

Photoacoustic Tomography (PAT) is a deep-tissue imaging modality, with potential clinical applications in the diagnosis of arthritis, cancer and other disease conditions. Here, we identified Clofazimine (CFZ), a red-pigmented dye and anti-inflammatory FDA-approved drug, as a macrophage-targeting photoacoustic (PA) imaging agent. Spectroscopic experiments revealed that CFZ and its various protonated forms yielded optimal PAT signals at wavelengths −450 to 540 nm. CFZ’s macrophage-targeting chemical and structural forms were detected with PA microscopy at a high contrast-to-noise ratio (CNR > 22 dB) as well as with macroscopic imaging using synthetic gelatin phantoms. *In vivo*, natural and synthetic CFZ formulations also demonstrated significant anti-inflammatory activity. Finally, the injection of CFZ was monitored via a real-time ultrasound-photoacoustic (US-PA) dual imaging system in a live animal and clinically relevant human hand model. These results demonstrate an anti-inflammatory drug repurposing strategy, while identifying a new PA contrast agent with potential applications in the diagnosis and treatment of arthritis.

The preclinical to clinical translation of new therapeutic and diagnostic agents is a very costly and high risk affair^1^,^2^. Because there are many uncertainties in the efficacy and safety profiles of new therapeutic agents, the development of drug repurposing and repositioning strategies for the clinical translation of established, FDA-approved drugs has experienced a boom during the past few years^1^,^3^. Usually, it takes more than a decade to get FDA-approval for a new chemical entity. Drug repositioning is revolutionising translational research by minimising the number of regulatory hurdles required to assure efficacy and safety, so the time for a new therapy to reach the clinic can be reduced to as little as three years^4^.

While drug repositioning strategies typically involve using an established therapeutic entity for treating patients with disease conditions outside the drugs’ intended therapeutic activity^1^,^5^,^6^,^7^, the potential of transforming drugs into bioimaging contrast agents for diagnostic applications remains unexplored. As most diseases stem from either acute or chronic inflammation caused by a variety of molecular, biochemical or physical factors^8^,^9^,^10^,^11^, an anti-inflammatory drug that can be re-formulated as a bioimaging agent could be tremendously beneficial in the clinic, either as a diagnostic tool or as a contrast agent to facilitate image-guided clinical interventions^12^. Independently, efforts aimed at exploiting immune cell-targeted diagnostic and therapeutic strategies^13^,^14^ prompted us to begin exploring the feasibility of repurposing a macrophage (MΦ)-targeted, biocompatible, non-toxic, photoacoustically active contrast agent from a pharmacopeia of FDA-approved, clinically used small-molecule anti-inflammatory drugs.

## Results

Photoacoustic (PA) detection relies on intrinsic absorption at specific excitation laser-generated wavelengths resulting in ultrasonic waves detected via conventional acoustic transducers^15^. For imaging applications, longer wavelengths are advantageous because they afford greater imaging depth, with reduced potential for phototoxicity^16^. Therefore, to search for candidate small molecule drugs with potential applications as PA contrast agents, we explored the optical properties of FDA-approved anti-inflammatory drugs. Every organic molecule has a characteristic optical absorption spectrum with coloured or pigmented compounds absorbing more light at higher wavelengths relative to colourless compounds^17^,^18^. After identifying drugs with the strongest optical absorbance and PA signals at visible wavelengths, we used PA-specific hardware to detect the drug in a variety of experimental and clinically-relevant platforms and tested their MΦ-targeting and anti-inflammatory capabilities in a relevant animal model.

### Screening FDA-Approved Anti-Inflammatory Drugs for Candidate PA Imaging Contrast Agents

**Figure 1:**
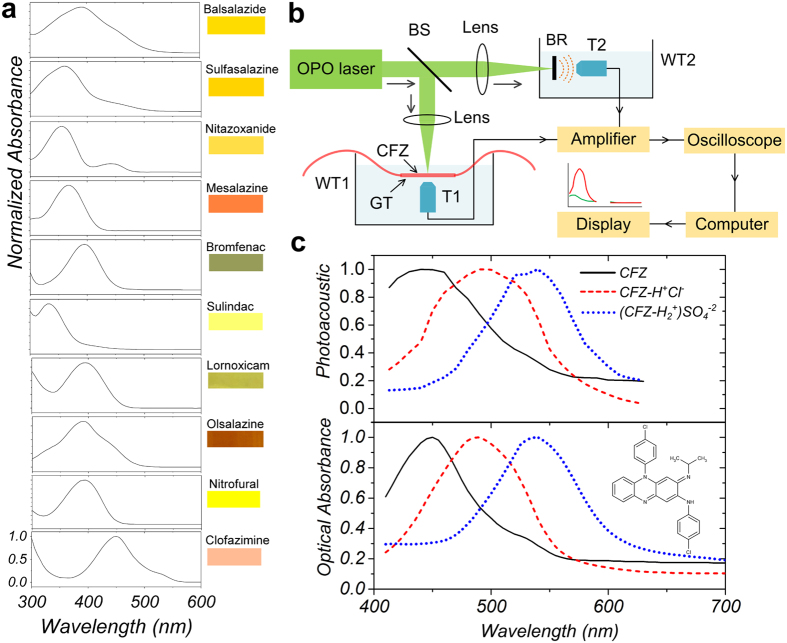
Screening for Candidate PA Imaging Contrast Agents. (**a**) Solution absorption spectra of the screened molecules (shown from 300 to 600 nm, at concentrations of 0.2–1 mM) when solubilised in DMSO. The colour of the drug is shown next to the spectra with the screened colour from the NPC plates (~10–20 mM) or from optical photography. (**b**) Schematic diagram for the setup for PA spectroscopy. OPO: Optical parametric oscillator; BS: beam splitter; BR: black rubber; WT1, WT2: water tanks; T1, T2: transducers; GT: glass tube (**c**) The solution absorption spectra and PA spectra of CFZ and its protonated forms in solution (Inset - chemical structure of clofazimine).

Currently, there are 1602 FDA-approved small molecule drugs on the market catalogued in the Drug Bank^19^. These drugs were sorted to focus on those with clinically established anti-inflammatory activity (126 in number, Supplementary Data File 1). In order to screen them for intrinsic colour with an optical absorption spectrum in the visible colour range or near IR range, these short-listed drug molecules were assayed using a combination of optical photography, colour identification, literature survey and high-throughput absorption spectrophotometry (Supplementary Data File 1). While the majority of the compounds were colourless, eight drugs were selected for further testing based on their optical properties while two other molecules were identified to be coloured via a parallel literature search (Supplementary Fig. S1, Supplementary Data File 1). Four of these 10 drugs showed high absorbance at wavelengths (*λ*) > 400 nm (Fig. 1a). Clofazimine (CFZ) exhibited the strongest and most red-shifted absorbance peak wavelength (*λ*_max_ = 450 nm) and piqued our interest as an established anti-microbial and anti-inflammatory drug specifically accumulating in MΦs – central regulators of the process of inflammation^20^,^21^,^22^,^23^.

### PA Spectroscopy of CFZ

To confirm CFZ’s potential use as a PA imaging agent, a PA spectroscopy unit was used to quantitatively establish the spectral properties of its PA signal, as computed via equation (1) (Fig. 1b). The measurements revealed wavelength-dependent variations in ultrasound wave generation corresponding to shifts in the absorbance spectra of CFZ when protonated (Fig. 1c, Supplementary Fig. S2). As a weakly basic drug possessing two protonatable amine groups, CFZ exhibited a pH-dependent, bathochromic shift of the PA spectra with maxima at λ_max_ = 450 nm, 495 nm and 540 nm. These three peaks in PA signal corresponded to the absorbance spectra of the free-base, monoprotonated and diprotonated molecular species, respectively^24^ (Supplementary Fig. S2).

### MΦ-targeting characteristics of CFZ

**Figure 2:**
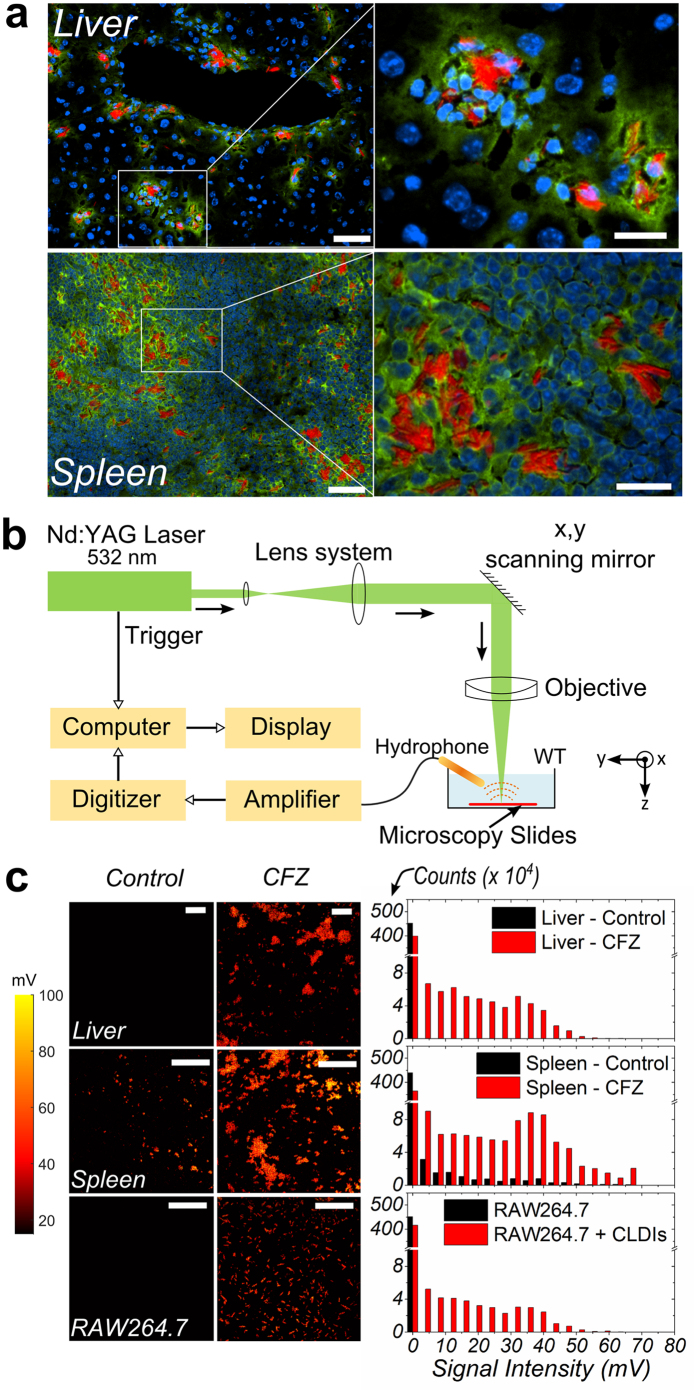
MΦ-Targeting and PA Microscopy of CFZ. (**a**) Cryosections of spleen and liver in 8-week CFZ-fed mice showed specific and massive accumulation of CFZ in CD68(+) MΦs. Green – CD68, Blue – nucleus, Red – CFZ. Scale bar = 50 μm (20 μm in digitally zoomed-in regions). (**b**) PA Microscopy Setup Schematic (WT-Water tank) (**c**) (*left*) PA microscopy of the (*top*) liver and (*middle*) spleen cryosections of vehicle and CFZ-fed mice and (*bottom*) RAW264.7 cells alone and incubated with CFZ inclusions (Scale bar = 50 μm). (*right*) Signal Intensity (mV) distribution across various samples (representative data from 3 acquired images).

Many animal models have been previously established to study the clinical pharmacology and pharmacokinetic properties of CFZ 20,25. Independently, to confirm the MΦ-associated accumulation of CFZ, we conducted immunofluorescence analyses using a positive MΦ marker (CD68)^26^ on histological sections of the spleen and liver from 8 week orally fed CFZ mice. Bright red pigmented spots, consistent with the natural colour of the drug, were observed in both the organs (Supplementary Fig. S3) which were also identified to be fluorescent in the far-red wavelength range^27^. As expected, these fluorescent red inclusions co-localised with CD68 expression in both the spleen and liver (Fig. 2a, vehicle-treated cryosections shown in Supplementary Fig. S4). This observation corroborates previous reports that CFZ does accumulate intracellularly as crystalline drug inclusions in the MΦs of various organs^21^,^28^ as reported in many preclinical animal studies as well as in human patients^20^,^29^,^30^.

### PA Microscopy of CFZ in MΦs

To quantitatively confirm and establish the PA signal intensity of these MΦ-containing CFZ inclusions, spleen and liver sections of CFZ-treated mice were imaged using a PA microscope using optical excitation at 532 nm (Fig. 2b). PA-responsive regions could be detected in liver (Contrast-to-Noise Ratio (CNR) = 22.4 dB (from equation (2)), signal intensity = 36.0 ± 12.5 mV) and spleen samples of CFZ-treated animals (CNR = 24.6 dB, signal intensity = 45.9 ± 21.2 mV) (red bars in Fig. 2c). Such regions were absent in liver samples of vehicle-treated animals while the spleen sections recorded low PA signal intensity in few areas of the tissue (CNR = 23.3 dB, signal intensity = 37.4 ± 18.5 mV) (black bars in Fig. 2c). To determine if MΦ associated CFZ inclusions found in the spleen were indeed the source of the PA signal, CFZ inclusions were isolated and purified from the spleen of CFZ treated animals^31^ and incubated with RAW264.7 cells *in vitro*. Following phagocytosis^31^, high PA signals were again detected in RAW264.7 MΦs containing the ingested CFZ inclusions (CNR = 26.7 dB, signal intensity = 29.1 ± 14.6 mV) while control RAW264.7 MΦs showed negligible PA signal intensity in the absence of any added CFZ (Fig. 2c).

### PA Imaging of CFZ in gelatin phantoms

**Figure 3:**
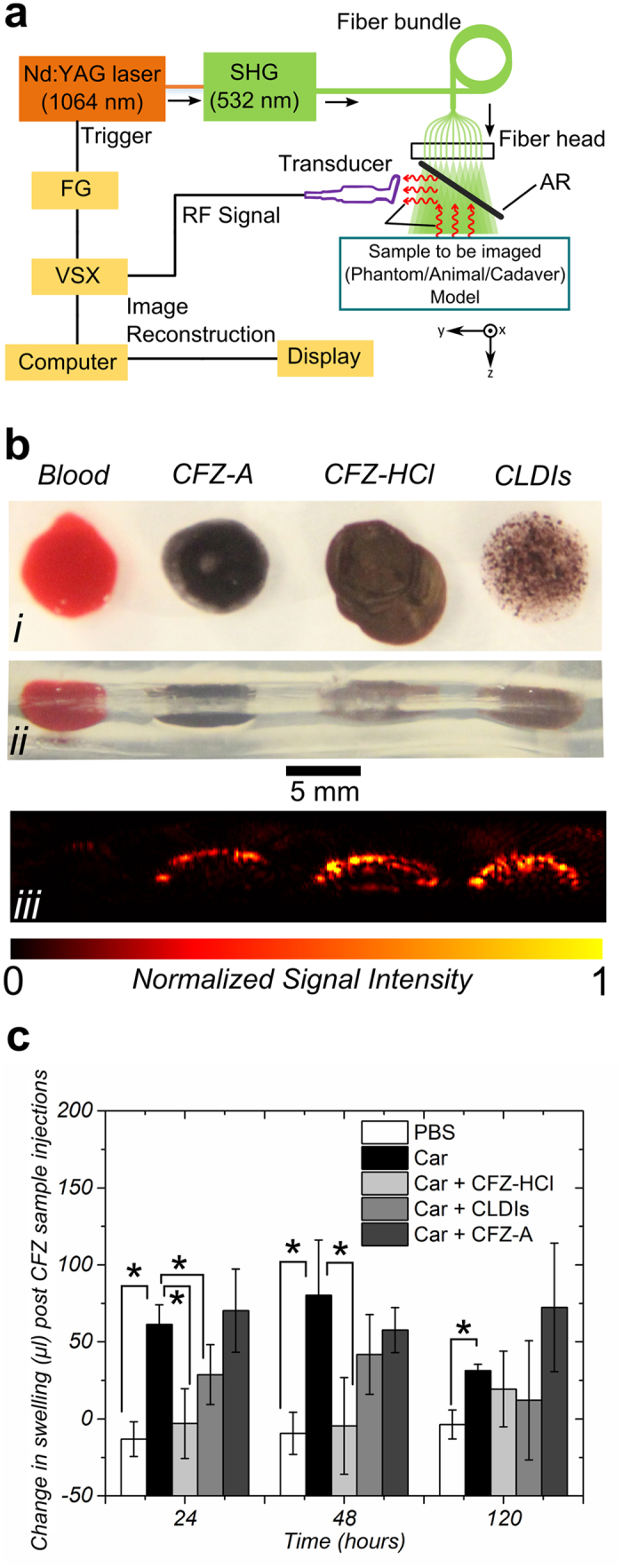
PA Imaging and Anti-Inflammatory Activity of CFZ. (**a**) Schematic of dual-modality PA-US imaging setup, (**b**) (*i*) top view, (*ii*) side view of phantoms loaded with whole human blood, CFZ-A, CFZ-HCl or CLDIs (all drug samples at 20 mM in DMSO or 20 mM soluble equivalent concentration resuspended in PBS), (*iii*) Normalised PA signals acquired and (**c**) Anti-inflammatory activity of CFZ as CFZ-A, CFZ-HCl or CLDIs (all at 2 mM in DMSO or 2 mM soluble equivalent concentration resuspended in PBS) in a carrageenan (Car)-based mouse footpad inflammation model (n = 4). Volume of the foot was calculated as per Supplementary Fig. S5. Edema (swelling) is reported as the change in volume with respect to volume measured just prior to CFZ sample injections. Statistical analysis was performed using a Student’s t-test (*p < 0.05).

A clinical imaging platform, combining dual modality imaging via PAT and ultrasound (US)^32^ (US-PA) was established to determine whether CFZ may be imaged in clinically relevant models (Fig. 3a). To target the translation from bench to bedside, sufficient imaging depth beneath the sample surface is usually desired. Photoacoustic Tomography (PAT) could offer much better imaging depth beyond the optical mean free path. To establish the suitability of CFZ for PAT, gelatin phantoms were prepared, embedded with sample wells containing whole human blood, soluble CFZ in monoprotonated form in DMSO (resolubilised CFZ-H^+^Cl^−^, hereby referenced as CFZ-A), CFZ inclusions isolated from the spleen of drug-treated mice (hereby referred to as Cystal-like-drug-inclusions or CLDIs) in PBS and their synthetic crystal analogue - CFZ-HCl microcrystalline particles in PBS^22^ (Fig. 3b). When visualised with PAT using an excitation wavelength of 532 nm, CFZ-A had a strong PAT signal while blood was minimally detected. Importantly, both synthetic CFZ-HCl crystals and mice-derived CLDIs exhibited significant PAT signals.

### Anti-inflammatory effect of CFZ

To confirm the *in vivo* anti-inflammatory activity of PA detectable forms of CFZ, an established carrageenan-based footpad-edema model^33^ (swelling measurement model shown in Supplementary Fig. S5) was used to test whether CFZ had a pro- or anti-inflammatory effect, after administration via different routes. Following 8 week oral treatment with CFZ, mice exhibited decreased inflammation upon stimulation with carrageenan at *t* = 4 h (~3-fold), 24 h (~14-fold) and 48 h (~2-fold) (*p < 0.05), compared to mice fed with a control drug-free diet (Supplementary Fig. S6). To test for therapeutic anti-inflammatory activity of local CFZ injections, naïve mice stimulated with carrageenan were re-injected locally in the footpad with CFZ-A, CFZ-HCl or CLDIs at *t* = 48 h post inflammation (Fig. 3c). Prior to re-injection, swelling was equivalent in all the animals (all *p* > 0.1) (Supplementary Fig. S7). Subsequent swelling in CFZ-HCl and CLDI injected footpads was inhibited for 24 h post therapy while the footpads in the carrageenan-alone treated animals continued to swell (*p* < 0.05) (Fig. 3c). The inhibition was sustained for a further 24 h in CFZ-HCl injected footpads (*p* < 0.05) (Fig. 3c). Interestingly, the anti-inflammatory activity was specific to the CFZ-HCl microcrystalline form of the drug, as injection of CFZ-A did not lead to a measurable anti-inflammatory response (Fig. 3c) (all *p* > 0.1). After 5 days, both treated and untreated animals recovered from their inflammatory injury, with the degree of swelling returning to normal levels (all *p* > 0.1) (Fig. 3c). Injection of CFZ-HCl or CLDIs in the absence of carrageenan, did not induce any measurable inflammation (Supplementary Figs S8 and S9). In contrast, injection of DMSO alone or in combination with CFZ-A resulted in a measurable inflammatory response initially (up to *t* = 24 h, *p* < 0.05) which declined by *t* = 96 h. (Supplementary Fig. S10).

### Dual-modality US-PA imaging in mice and human clinical model

**Figure 4:**
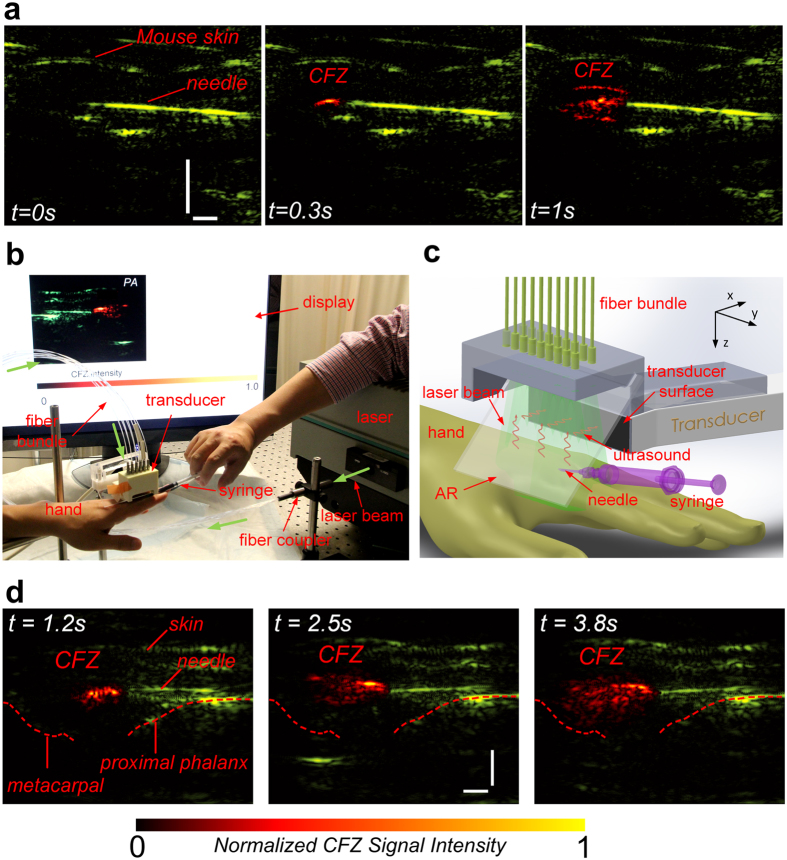
Mouse and Clinical Finger-Joint Imaging. (**a**) PA images obtained of injecting CLDIs into the posterior peritoneal cavity of the mouse. CLDIs were injected into the joint through the needle with the injection lasting for ~1 s, during which both PA and US images (Supplementary Fig. S11) were continuously recorded. In comparison with the image taken at time *t* = 0 s (shown in green), the PAT signal of CFZ (coloured in red) at *t* = 0.3 s and 1 s clearly show the appearance and distribution of CFZ, (**b**) Photograph of the dual modality PA-US imaging setup for clinical imaging, (**c**) Close-up model of the injection site and imaging probe and (**d**) PA-US (background) images obtained of CFZ-A injected into the human cadaver MCP joint of the index finger. CFZ-A was injected into the joint through the needle with the injection lasting for ~4 s, during which both PA and US images were continuously recorded. Three representative snapshots of the process are shown. In comparison with the image taken at time *t* = 0 s (shown in green), the PAT signal of CFZ (coloured in red) at *t* = 1.2 s, 2.5 s and 3.8 s clearly show the appearance, development and dispersion of CFZ in the injected joint (corresponding movie – Supplementary Movie 1). Scale bars – vertical = 3 mm, horizontal = 2 mm.

To assess the suitability of CFZ as a PA contrast agent, 10 week-old mice were used to monitor if CFZ could be imaged under *in vivo* conditions (Fig. 4a, Supplementary Fig. S11). In comparison with the PA image taken just before injection (at *t* = 0 s, shown in green, US image in Supplementary Fig. S11), the images taken at *t* = 1.0 s and 1.2 s clearly show the appearance and increase of a PAT signal attributed to CFZ. To further determine whether CFZ may be useful as a contrast agent for the diagnosis or treatment of arthritis, the metacarpophalangeal (MCP) joint in the index finger of the hand of a human cadaver (joint usually affected by inflammatory arthritis) was selected as the site of injection of CFZ (Fig. 4b,c). In comparison with the image taken just before injection (at *t* = 0 s, shown in green, Supplementary Fig. S12), the images taken at *t* = 1.2 s, 2.5 s and 3.8 s clearly show the appearance and increase of the PAT signal of CFZ in the joint (shown as red in Fig. 4d, Supplementary Movie 1). While US images show the relevant anatomical features – mouse posterior in the *in vivo* mouse imaging and the proximal phalanx and the metacarpal forming the MCP joint in the clinical human hand imaging (Supplementary Fig. S11 and S12), PAT, benefitted by the good spatial resolution and high sensitivity to the strong optical absorption of CFZ, shows the advective transport of the drug upon injection.

## Discussion

The repurposing of older, approved drugs for newer therapeutic applications is an emerging, important area of translational research. *In silico* approaches, typically relying on literature analyses^3^,^34^, computational screening^5^,^35^ and high throughput experimental screening^6^,^7^ have been successful in terms of identifying new potential applications for existing drugs. Furthermore, large federally-supported research programs have been established by the National Center for Advancing Translational Sciences (NCATS) with the purpose of supporting atypical, translational drug development approaches that can complement the traditional bench-to-bedside research approaches that are typically pursued by the pharmaceutical industry^36^.

Using these tools and resources, we have identified a new candidate PA contrast agent from a collection of FDA-approved anti-inflammatory drugs that were screened for favourable optical properties in clinical applications^37^,^38^ (Fig. 1). As a lead drug candidate for formulation and further clinical development, CFZ showed excellent optical signals when probed using PA spectroscopy. CFZ’s MΦ-targeting capability, associated with the formation of unique intracellular crystallized inclusions (CLDIs), was confirmed via conventional immunofluorescence assays and detected using a PA microscope (Fig. 2). CLDIs and their associated synthetic soluble (CFZ-A) and structural (CFZ-HCl) forms were subjected to additional imaging and *in vivo* anti-inflammatory models^22^ wherein all three forms showed excellent PA detectability (Figs 3 and 4) and CFZ-HCl and CLDIs displayed significant anti-inflammatory activity as also shown in prior studies using RAW264.7 cells^31^ (Fig. 3).

The molar extinction coefficient *ε* of oxygenated and deoxygenated hemoglobin at 532 nm is 43876 cm^−1^M^−1^ and 40584 cm^−1^M^−1^, respectively. At the concentration of ~5 mM as in a physiological environment, the absorption coefficient (*μ*_*a*_) of fully oxygenized blood and fully deoxygenized blood are 219.38 cm^−1^ and & 202.92 cm^−1^, respectively^39^. In comparison, *ε* for CFZ-H^+^ is 16945.34 ± 2750.46 cm^−1^M^−1^ (n = 3). At a concentration of 20 mM as used in our experiment on phantoms, the absorption coefficient *μ*_*a*_ for CFZ-H^+^ at is 338.91 cm^−1^ which offers a higher contrast and stronger PA signal over blood in the background. Moreover, the drug inclusions of CFZ (CLDIs) and CFZ-HCl consist of CFZ-H^+^ as a chloride salt with concentrations reaching up to 3 M within the drug crystal^22^. This implies that the MΦ-targeted drug precipitates can have a maximum *μ*_*a*_ of 50836.02 cm^−1^ at 532 nm in theory (~200-fold higher than blood). Thus, extremely high PA activity resulting in precise monitoring of MΦs in relation to blood is possible via formulation of CFZ as solid, therapeutic nano and microcrystals of protonated CFZ salts.

Indeed, one of the key cellular effectors of inflammation are MΦs. MΦs are vital for mammalian self-nonself recognition and immunological homeostasis, particularly for resolving the inflammatory response^40^,^41^,^42^. Recent evidence also indicates that dysregulation in MΦ signaling responses can lead to inherently deleterious effects on the host with pathological consequences resulting in arthritis^11^,^43^, tumor growth and metastasis^44^,^45^, atherosclerotic plaque formation^46^, diabetes^47^ and other disease conditions. Thus, MΦ-targeted drugs and bioimaging contrast agents offer great promise for the diagnosis and treatment of inflammatory diseases, infections and cancers^12^. Beyond simple diagnosis, theranosis –the combination of diagnosis and therapy – is also being sought as a means to develop highly personalised therapeutic approaches^48^.

In terms of clinical significance, arthritis and other rheumatic diseases remains a leading cause of disability among adults in the U.S. and is associated with decreased work productivity, reduced quality of life, and high health-care costs (upto US$128bn, for rheumatoid arthritis alone – ~27k per patient per year in 2012)^49^. To facilitate disease diagnosis and early therapeutic interventions, the National Institute of Arthritis and Musculoskeletal and Skin Diseases (NIAMS) has called upon the scientific community to develop early detection technologies via modern medical imaging technologies such as PAT, that can be used to visualise soft tissue structure and function in the joints^50^, so as to improve upon current diagnostic procedures such as MRI and X-rays^51^. Furthermore, a more localised, targeted approach for diagnosis-assisted treatment of arthritis would be highly beneficial to patients, since state-of-the-art, anti-inflammatory therapies are mostly designed to act systemically, leading to significant, off-target side effects. CFZ’s high MΦ-specificity (>95%) is expected to provide MΦ-associated PA activity enabling optimal soft-tissue detection in inflammation. While the drug flow-rate was observed to be high in the human hand model, dictated primarily by the injection velocity, it is expected that in an inflamed joint CFZ will be immediately internalized by MΦs. As such, due to the long half-life of CFZ and high solid-state stability as non-toxic crystals, CFZ formulations could be designed for long-term monitoring of the joint via a single injection alone.

To conclude, repurposing CFZ as an MΦ-targeted PA agent is highly attractive, because of the drug’s well documented preclinical and clinical anti-inflammatory properties, its affinity for MΦs, as well as its favourable optical characteristics, biocompatibility, and its suitability for multimodal (US/PA/fluorescence) imaging^27^. Since CFZ accumulates massively inside MΦs without apparent toxicity, and because of the high stability of intracellular CFZ crystals, there also exists significant potential in terms of optimising CFZ as a potent long-term, intra-articular anti-inflammatory theranostic formulation, to provide a highly localised, site-directed, individualised therapy for arthritic patients, without the side effects of systemic anti-inflammatory therapies^43^,^52^,^53^,^54^.

## Methods

### Screening of FDA-approved drugs

The initial screening to obtain a short-list of drugs from Drugbank was done via Boolean search to extract all small molecule drugs that were FDA-approved, not withdrawn nor illicit and containing the descriptive term “anti-inflammatory” resulting in a short list of 126 molecules. Two 1536-well plates containing a common subset of the NCGC Pharmaceutical Collection (NPC)^55^ were selected to be analysed for visible colour. The plates included 87 short-listed drugs solubilised in dimethyl sulfoxide (DMSO) up to a concentration of 20 mM (Supplementary Data File 1) that were then photographed using a flatbed scanner and measured for their UV/Vis absorbance profiles. The raw PNG formatted image was manually cropped and stored as 2 separate PNG images. A simple custom Java program was then used to select the centremost pixel from each well and report the colour information in both RGB and HSV format. Molecules not yet available in NPC format (39 in number) were searched in literature, USP Pharmacopeia and other databases for their colour and absorption spectra (their colours are indicated in Supplementary Data File 1). To re-evaluate the UV/Vis profile of the 10 pigmented compounds, samples were prepared at a concentration of 0.2–1 mM in DMSO and spectra was recorded from 300–1000 nm on a Biotek II multi-well plate reader. The solution absorbance was normalised with the corresponding value using a control sample containing DMSO alone and plotted from 300 to 600 nm as no discernible absorbance was measured for any of these compounds at >600 nm.

### PA Spectroscopy and Sample Preparation

The setup for PA spectroscopy measurement is shown in Fig. 1b. The laser source is a tunable OPO (Surelite OPO Plus, Continuum) pumped by the third harmonic of an Nd:YAG laser (Surelite, Continuum). The OPO laser, working at a repetition rate of 10 Hz, can provide a super broad tuning range of 410–650 nm, 710–2500 nm. The emitted laser was separated into two parts by a beam splitter (BS) with a splitting ratio of 90:10. The reflected beam was focused on the sample by a lens. The sample was placed in a transparent glass tube (Kimble Chase North America) with an inside diameter 1.1 mm and a wall thickness 0.2 mm, which was submerged in a water tank (WT). The transmitted beam was focused on a black rubber, which could absorb all incident laser energy and was used as a reference for laser pulse energy calibration. The generated PA signals from the sample and the black rubber were received by two transducers T1 and T2 (C323, Olympus NDT), amplified by a pulser and receiver (5072R, Olympus NDT), digitised by an oscilloscope (TDS 540A, Tektronix) and finally collected by a computer. PA signal intensity (*p*) at a specific wavelength *λ* is linearly proportional to the corresponding absorption coefficient *μ*_*a*_(*λ*) of the sample. Mathematically, this can be formulated as


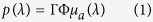


where Γ is the Grüneisen parameter, which is a constant and Φ is the laser fluence, which was calibrated using the reference black body^56^. Clofazimine (CFZ) as a free base was obtained directly from Sigma-Aldrich (St. Louis, MO, Catalogue No. C8895) and solubilised in DMSO and confirmed via ^1^H-NMR to be unprotonated in solution^22^. To obtain CFZ-H^+^Cl^−^ in solution, CFZ-HCl crystals, obtained via crystallisation at pH 5 as published before^22^, were resolubilised in DMSO. Briefly, equal volumes of 1 M NH_4_Cl in H_2_O and 2 mM CFZ in methanol were mixed with the addition of a surfactant (up to 2% (v/v) Triton^TM^ X-100, Sigma-Aldrich, Catalogue No. X100) and incubated at room temperature for 48 hours. The precipitates obtained were washed twice with H_2_O and lyophilized followed by storage at −20 °C until used. Just prior to analysis, CFZ-H^+^Cl^−^ crystals were solubilised in DMSO. These solubilised samples were also confirmed to be monoprotonated via ^1^H-NMR. For diprotonated CFZ, free-based CFZ crystals were dissolved in 9 M H_2_SO_4_ resulting in the formation of a deep purple coloured solution of diprotonated CFZ (Supplementary Fig. S2).

### *in vivo* CFZ model for oral feeding

Experiments using the CFZ mice model were performed as published before^21^,^22^,^28^. Mice (4 week old, male C57Bl/6) were purchased from the Jackson Laboratory (Bar Harbor, ME) and acclimatised for 2 weeks in a specific-pathogen-free animal facility. All animal care was provided by the University of Michigan’s Unit for Laboratory Animal Medicine (ULAM). The experimental protocol was approved by the Committee on Use and Care of Animals and all experiments were carried out in accordance with the approved guidelines. CFZ was dissolved in sesame oil (Roland, China, or Shirakiku, Japan) to achieve a concentration of 3 mg/ml, which was mixed with Powdered Lab Diet 5001 (PMI International, Inc., St. Louis, MO) to produce a 0.03% drug to powdered feed mix. A corresponding amount of sesame oil was mixed with chow for vehicle treatment (control). See Supplementary Document for preparation of microscopy slides and immunofluorescence assay.

### *in vitro* cell-line experiments

The murine MΦ cell line RAW264.7 was purchased from ATCC (Manassas, VA) and maintained in growth media (DMEM (Life Technologies, Carlsbad, CA) supplemented with 10% FBS and 1% penicillin/streptomycin). Isolated CLDIs were added at 20 μM solution equivalent concentration in growth media to 8-well chamber-slides (Nunc^TM^ Labtek^TM^ II, Thermo-Scientific Catalogue No. 154534) containing 5 × 10^4^ RAW264.7 cells/well and incubated (24 h at 37 °C and 5% CO_2_). Following incubation, cells were fixed using 4% paraformaldehyde in PBS for 15 mins at room temperature followed by rinsing and washing with ice-cold PBS twice. The samples were counterstained with Hoechst33342 for nuclear labelling followed by removal of chambers. A drop of Prolong Gold® (Life Technologies) was placed on the sample and covered with a glass coverslip for imaging.

### PA Microscopy Setup

The setup for PA microscopy measurement as described previously^57^ is shown in Fig. 2b. The laser source is a diode-pumped solid-state Nd:YAG laser (Spot-10-200-532, Elforlight Ltd, UK), with a wavelength of 532 nm and a pulse duration of 2 ns. The emitted laser was first collimated by a lens system, then reflected by a two-dimensional (x, y) scanning mirror (6230H, Cambridge Technology), and finally, focused on the sample by an achromatic objective (AC254-040-A, ThorLabs) with a focal length of 40 mm. The microscopy slides or chambers submerged in phosphate buffered saline (PBS) buffer, absorbed the green laser and produced acoustic signals. The generated signals were captured by a hydrophone (centre frequency-35 MHz, 6 dB bandwidth 100%), amplified by a low-noise amplifier (ZFL-500LN, Mini-Circuits), digitised by an A/D card (Cobra CompuScope CS22G8, GaGe), transferred to the computer, and finally reconstructed (as signals in mV) using the maximum amplitude projection (MAP) algorithm for visualisation^58^. The calibrated lateral resolution of the system was 2 μm. The contrast-to-noise ratio (CNR) of the images was calculated by the expression


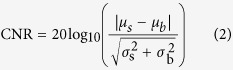


where *μ*_*s*_ and *μ*_*b*_ are the means of the signals in the region of interest (ROI) and the background respectively and *σ*_*s*_ and *σ*_*b*_ are the corresponding standard deviations. Signal intensity histograms of the acquired images were constructed via measurements in ImageJ (National Institutes of Health, Bethesda, MD).

### Mice Carrageenan Footpad Edema Model

Carrageenan (C1867, Sigma-Aldrich^®^, 2% in PBS, 30 μl) was injected into the right footpad while PBS (30 μl) was injected in the left footpad as a negative control. For preliminary testing of CFZ as an anti-inflammatory agent, 8-week orally fed mice (CFZ and control) were used. For testing of CFZ in soluble and microcrystal form, naïve 4–5 week old mice were injected with carrageenan first followed by injection of CFZ-A (2 mM in DMSO, 30 μl), CLDIs (2 mM equivalent in PBS, 30 μl) or CFZ-HCl (2 mM equivalent in PBS, 30 μl) at 48 h post carrageenan injection. For control experiments, CFZ-A, CLDIs and CFZ-HCl were injected into the right footpads of naïve 4–5 week old mice. DMSO was used as a control for CFZ-A in the left footpad whereas PBS was used as a control for CLDIs and CFZ-HCl. Footpads were measured (length - *l*, width - *w*, three thicknesses – *t*_*1*_, *t*_*2*_, *t*_*3*_) at various time points using a digital vernier calliper and changes in the swelling based on volume of the foot was calculated as per Supplementary Fig. S5.

### Synthesis of Gelatin Phantoms

8% gelatin from porcine skin (G2500, Sigma) was dissolved in water, and then heated and stirred on a hotplate for about one hour. After cooling down, several wells of equal volume were dug out of the surface and filled with CFZ samples or human whole blood (requested from the Department of Pathology, University of Michigan). After that, smaller 8% gelatin phantoms was used to cover the surface of the previous one to seal the embedded samples.

### Clinical human model experiments

A cadaver hand was requested from Anatomical Donation Program at University of Michigan through an organ donation program. Once received, the cadaver tissue was frozen in a lab specimen freezer under −20 °C. Before the imaging experiment, it was submerged in flowing cold water and thawed for about one hour. The request, transportation, storage, and handling of the cadaver tissue followed the policies and guidelines of Michigan Anatomical Gift Law Public Act 368 of 1978, amended as Public Act 39 of 2008. After the experiment, the cadaver tissue was returned to the Anatomical Donation Program.

### PA Tomography of Synthetic Phantoms, Mouse Model and Cadaver hand

The details of the hardware used have been reported in previous publications^32^,^59^ and a schematic and setup is shown in Figs 3a and 4b. The dual-modality platform was built based on the commercial Verasonics® (VSX) system (Vantage 256, Verasonics). In the PA imaging configuration, the second harmonic output (i.e., 532 nm) of an Nd:YAG laser (Powerlite DLS 8000, Continuum) with a pulse duration of 6 ns and a repetition rate of 10 Hz was used as the laser source. Ultrasound gel (MediChoice, The Medical Supply Group, Atlanta, GA) was topically applied for better acoustic matching between the transducer and the samples. The emitted laser was delivered to the sample through an optical fiber bundle. The excited PA waves from the sample, which propagated through ultrasound coupling gel, was reflected by a transparent glass slide (acoustic reflector, AR) and then received by a transducer array (L7-4 for phantom and CL15-7 for cadaver tissue, Philips Healthcare). The captured radio-frequency (RF) signals were amplified and digitised by the Verasonics system, then transferred to the computer for reconstruction and visualisation. Since a graphics processing unit (GPU) was employed to facilitate parallel computation, the dual-modality system can acquire, reconstruct and display two-dimensional PA images with a frame rate up to 10 Hz, which is only limited by the pulse repetition rate of the laser. In US imaging configuration, the Verasonics system drives the transducer array to emit ultrasound waveforms and receive echoes to form ultrasound images. The acquired RF signals were transferred to the computer, reconstructed using MATLAB® using previously published algorithms^60^ and displayed on the monitor. A function generator (FG, 3314A, Hewlett-Packard) was used to generate separate trigger signals for PA imaging and US imaging to ensure the correct timing. For mouse imaging, a 23G syringe needle with a nominal outer diameter of 0.64 mm was inserted into the posterior section of the peritoneum in close proximity to the hind limbs (Supplementary Fig. S11). CLDIs at 20 mM suspended in PBS solution was injected slowly into the area via the inserted syringe needle. For the clinical human model imaging, a 23G syringe needle with a nominal outer diameter of 0.64 mm was inserted into the target metacarpophalangeal (MCP) joint of the cadaver hand transcutaneously, guided by the US-PA dual-modality imaging system with its tip stopped next to the joint about 5.6 mm beneath the skin surface. A commercial ultrasound machine (z.one ZONARE) equipped with a linear probe (L10-5, ZONARE) was used to further confirm the positioning of the needle in the joint. CFZ-A at 5 mM in DMSO solution was injected slowly into the joint via the inserted syringe needle. For all phantom, mice and human model experiments, the ROI and background in the reconstructed PA images were segmented and coloured based on different colour schemes for the purpose of illustration. The laser fluence on the skin surface was estimated to be 4 mJ/cm^2^, which is well below the American National Standards Institute (ANSI) safety limit at 532 nm (as per ANSI Z136.1, Laser Institute of America). The intensities of the injected solutions were normalised.

## Additional Information

**How to cite this article**: Keswani, R. K. *et al*. Repositioning Clofazimine as a Macrophage-Targeting Photoacoustic Contrast Agent. *Sci. Rep.* **6**, 23528; doi: 10.1038/srep23528 (2016).

## Supplementary Material

Supplementary Information

Supplementary Data File 1

Supplementary Movie 1
