# Supplementary material for: Repositioning Clofazimine as a Macrophage-Targeting Photoacoustic Contrast Agent
Source: Sci Rep. 2016 Mar 22;6:23528. doi: 10.1038/srep23528 (PMC4802322; doi:10.1038/srep23528)
Supplement: Supplementary Information [file srep23528-s1.pdf]

SUPPLEMENTARY INFORMATION

Repositioning Clofazimine as a Macrophage-Targeting Photoacoustic Contrast Agent

Authors:

Rahul K. Keswani[1,†], Chao Tian[2,4,†], Tyler Peryea[3], Girish Gandikota[2], Xueding Wang[2,4,*], Gus R. Rosania[1,*]

Supplementary Data

Supplementary Data File 1. List of 126 FDA-approved small molecule drugs with previously reported anti-inflammatory properties and their pigment colour (in .xlsx format)

Supplementary Movie

Supplementary Movie 1. Injection of CFZ into the metacarpophalangeal joint (MCP) monitored via PA-US dual imaging system

Supplementary Materials and Methods

Microscopy Specimen Preparation. Post 8 weeks of drug feeding, mice were euthanised by ketamine/xylazine intraperitoneal injections followed by exsanguination. Spleen and liver organs were isolated and immediately submerged in optimal cutting temperature compound (Tissue-Tek Cat. no. 4583; Sakura), and frozen (-80°C). Cryosectioning was carried out using a Leica 3050S cryostat (thickness - 10 μm) and mounted onto a glass slide. Spleens were harvested and CLDIs were isolated using a previously described method[31]. Briefly, the spleens were cut into small pieces, homogenised with a syringe plunger, and then filtered through a 40 μm cell strainer to remove connective tissue debris. The spleen homogenate was centrifuged (300 ×*g* for 10 min) to remove large cell debris and the pelleted CLDIs were resuspended in 10% sucrose in Dulbecco's PBS (DPBS). CLDIs were further purified using a three-layer discontinuous gradient (50%, 30%, and 10% sucrose in DPBS) centrifugation method (3200 ×*g* for 30 min). CFZ content was spectrophotometrically measured using a previously described method using 9 M H_2SO_4 as a solvent with absorbance readings at λ=540 nm[31].

Immunofluorescence assay. Microscopy slides of spleen and liver cryosections were fixed using 4% paraformaldehyde in PBS for 15 mins at room temperature followed by rinsing and washing with ice-cold PBS twice. The samples were permeabilised using 0.25% Triton X-100 in PBS for 10 mins at room temperature followed by rinsing and washing with ice-cold PBS thrice. The samples were then incubated with blocking buffer (0.05% Tween 20, 1% Bovine Serum Albumin, 5% goat serum, 0.3 M glycine in PBS) for 30 mins at room temperature. The samples were then incubated with 0.2 ml of 1:100 dilution of primary anti-CD68 antibody (abcam® ab53444) overnight at 4 °C followed by washing with ice-cold PBS thrice. Fluorescence staining was performed by incubating with 1:200 dilution of secondary antibodies (IgG (H+L) Alexa Fluor® 488, Cell Signaling® 4416S) for 1 hour at room temperature followed by washing with ice-cold PBS thrice. The samples were counterstained with Hoechst33342 for nuclear labelling followed by removal of chambers. A drop of Prolong Gold® (Life Technologies was placed on the sample and covered with a glass coverslip for imaging.

Supplementary Figures

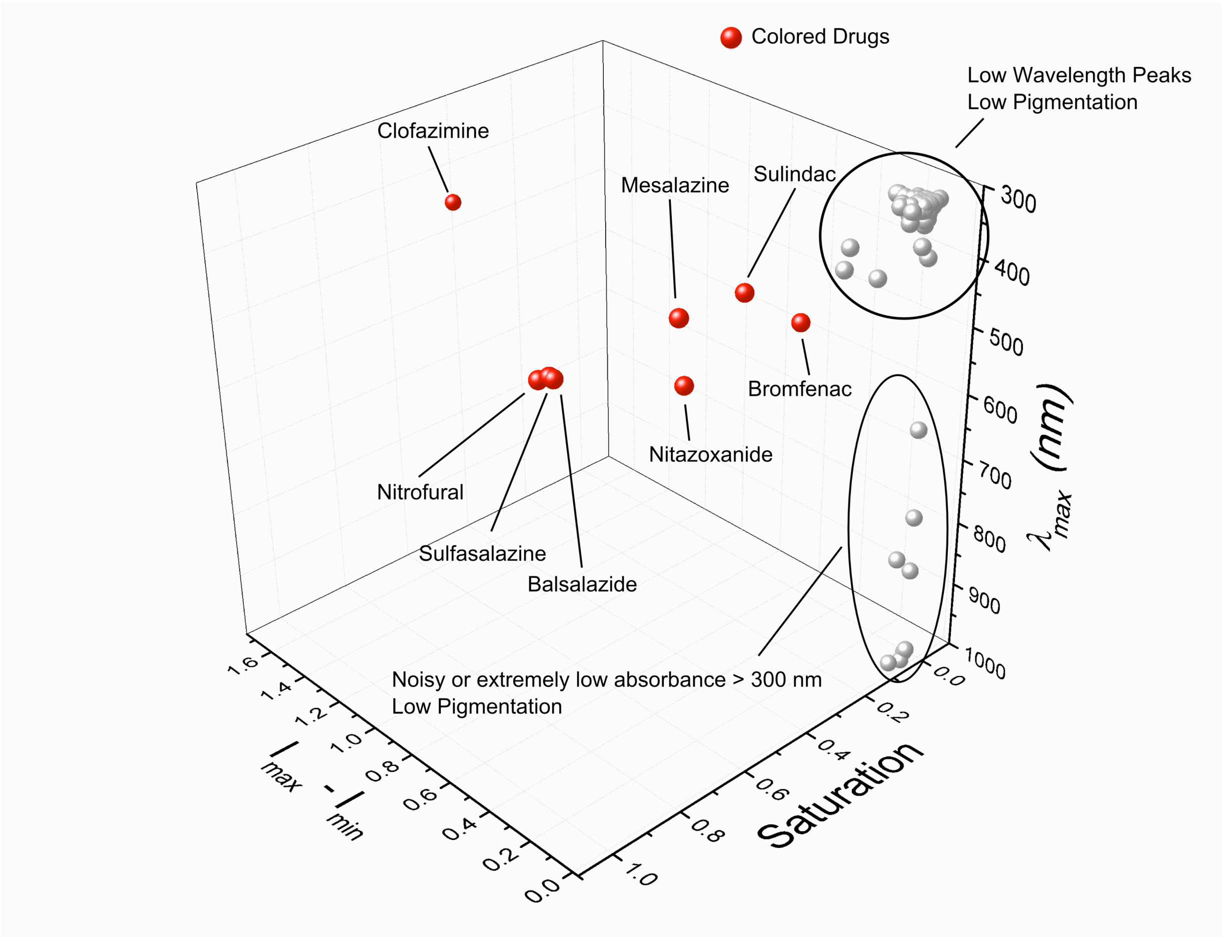

Supplementary Figure S1. Pigmented Drug Profiling. Identification of pigmented anti-inflammatory drugs via Saturation (S in HSY extracted from RGB of colours photographed on NPC plates), absorption spectrophotometry profile as maximum intensity difference ($I_{max} - I_{min}$) and wavelength of maximum absorption (λ_{max}). Pigmented drugs had high saturation, higher λ_{max} (>350 nm) and higher intensity differences.

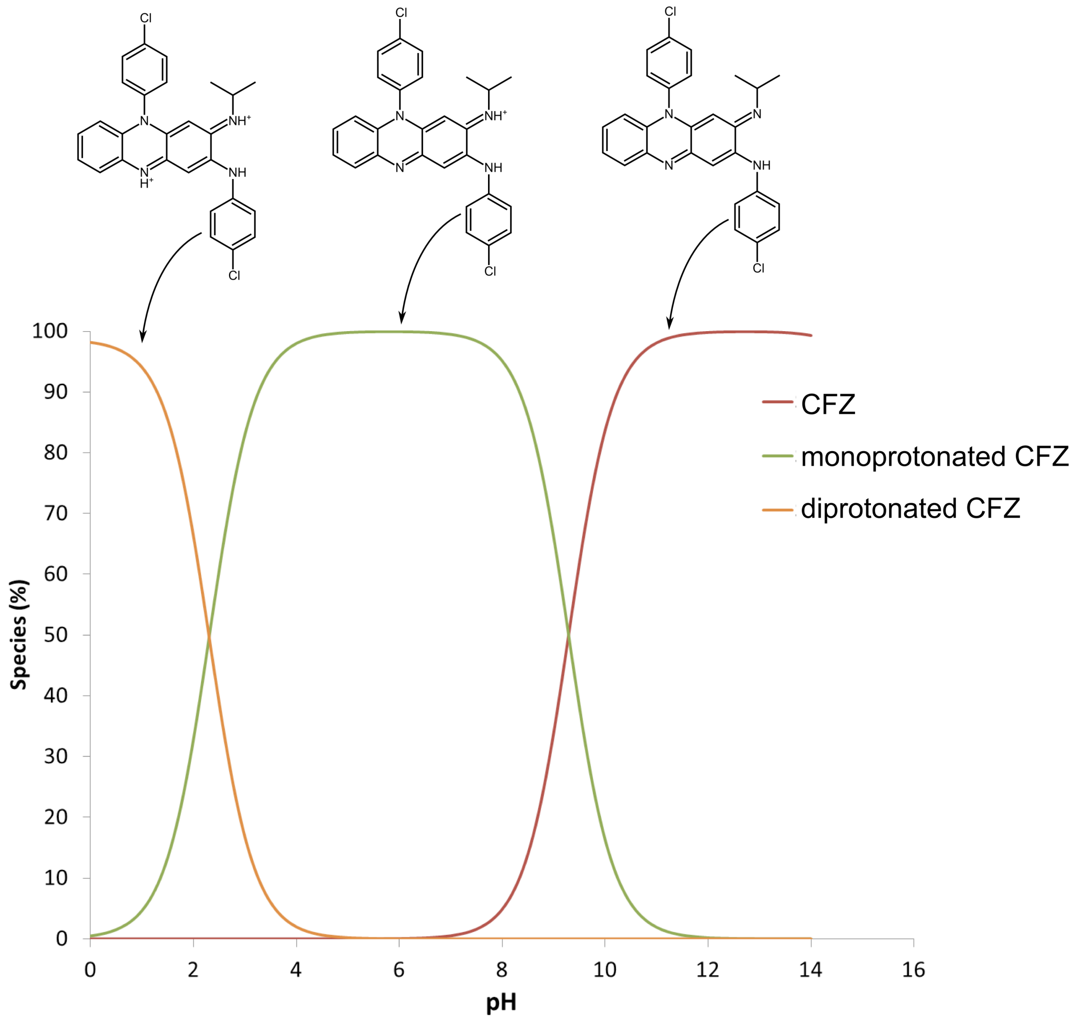

Supplementary Figure S2. Prediction of CFZ solution forms via ChemAxon. Protonation profile of CFZ under variation of pH in accordance with its pK_a.

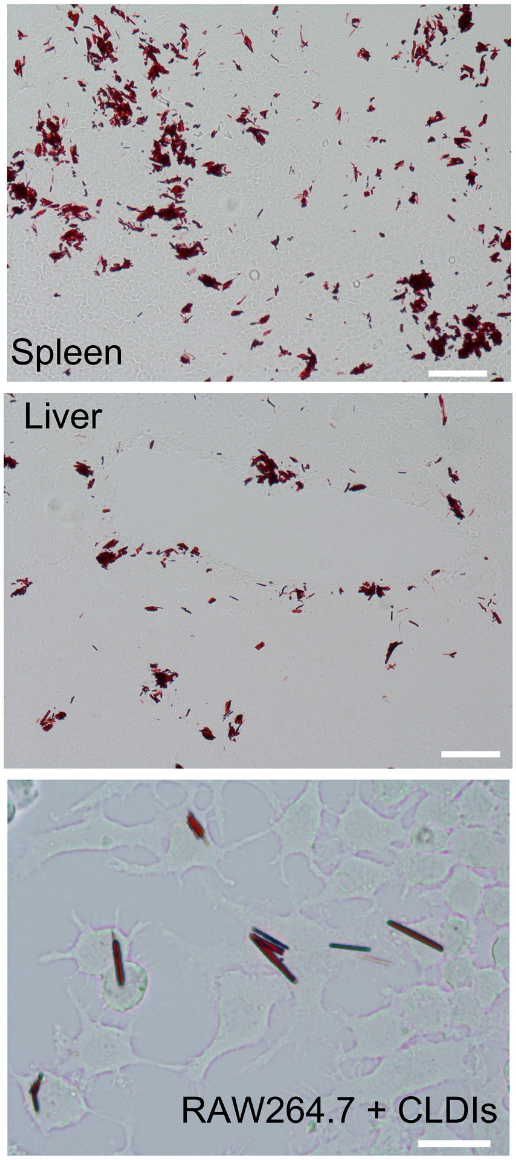

Supplementary Figure S3. Brightfield Imaging of Clofazimine Accumulation. (*top*, *middle*) Cryosections of spleen and liver in 8-week CFZ-fed mice imaged using conventional optical microscopy showing bright red inclusions of CFZ. Scale bar = 50 µm. (*bottom*) Spleen isolated CFZ inclusions (CLDIs) present within RAW264.7 MΦs. Scale bar = 20 µm.

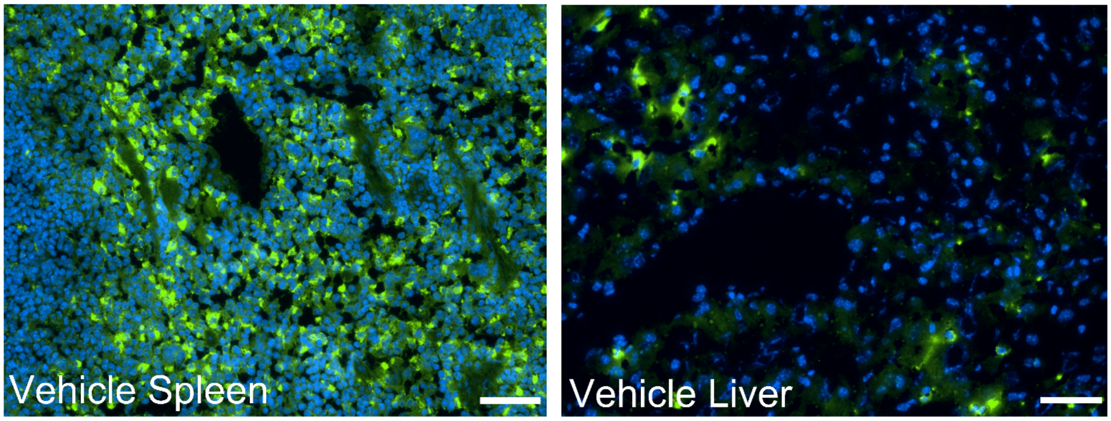

Supplementary Figure S4. Vehicle Liver and Spleen Immunostaining. Cryosections of spleen and liver in 8-week control-fed mice immunostained using fluorescent markers showing inherent CD68(+) MΦs. Green – CD68, Blue – nucleus. Scale bar = 50 μm.

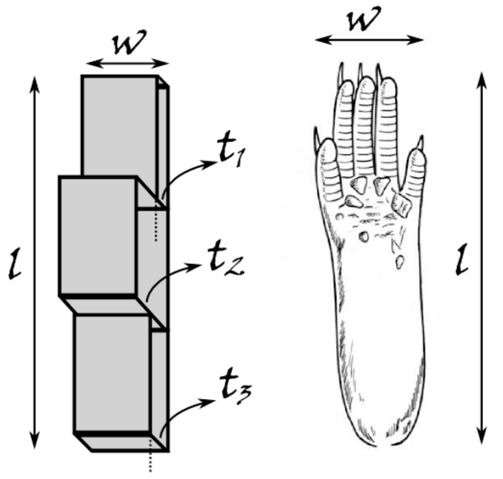

$$V = w.(l/3).(t_1 + t_2 + t_3)$$

Supplementary Figure S5. Edema (swelling) calculations. Calculation of footpad volume based on measured dimensions.

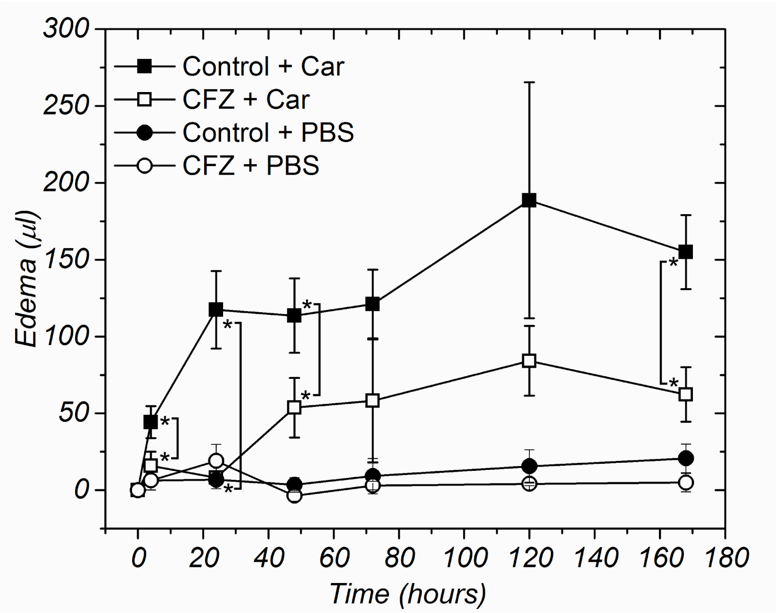

Supplementary Figure S6. Anti-inflammatory activity of orally-fed CFZ. 8 week CFZ-fed and Control-fed mice (n=4) were injected with carrageenan (Car, 2% in PBS, 30 µl) in the right footpad and with PBS (30 µl) in the left footpad at time *t*=0 h and the swollen volume (edema) was calculated as shown in **Supplementary Fig. S5**. CFZ-fed mice had lower swelling at *t*=4 h (~3-fold), 24 h (~10-fold), 48 h (~2-fold) and 168 h (~2.5-fold) post injection (*, p<0.05).

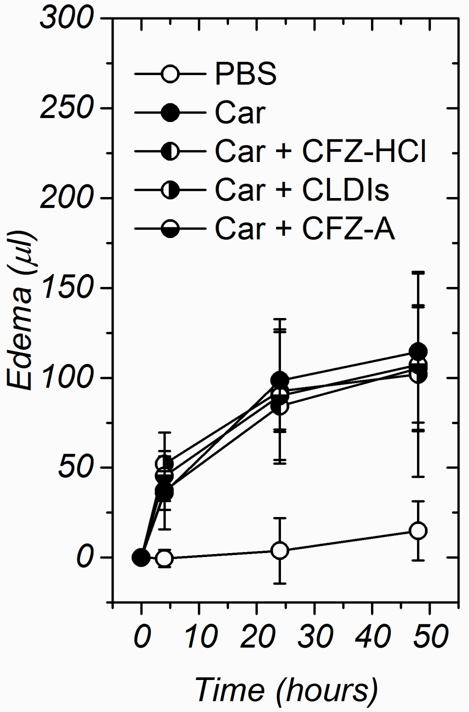

Supplementary Figure S7. Effect of carrageenan on footpad swelling prior to CFZ injections. Mice (n=4) were injected with carrageenan (Car, 2% in PBS, 30 µl) in the right footpad and with PBS (30 µl) in the left footpad at time t=0 h and the swollen volume (edema) was calculated as shown in **Supplementary Fig. S5**. Carrageenan injected footpads showed significant swelling relative to PBS injected footpads (all $p<0.05$) and equivalent swelling across the three subsequent groups used for CFZ injections ($p>0.10$ at all time-points relative to each other).

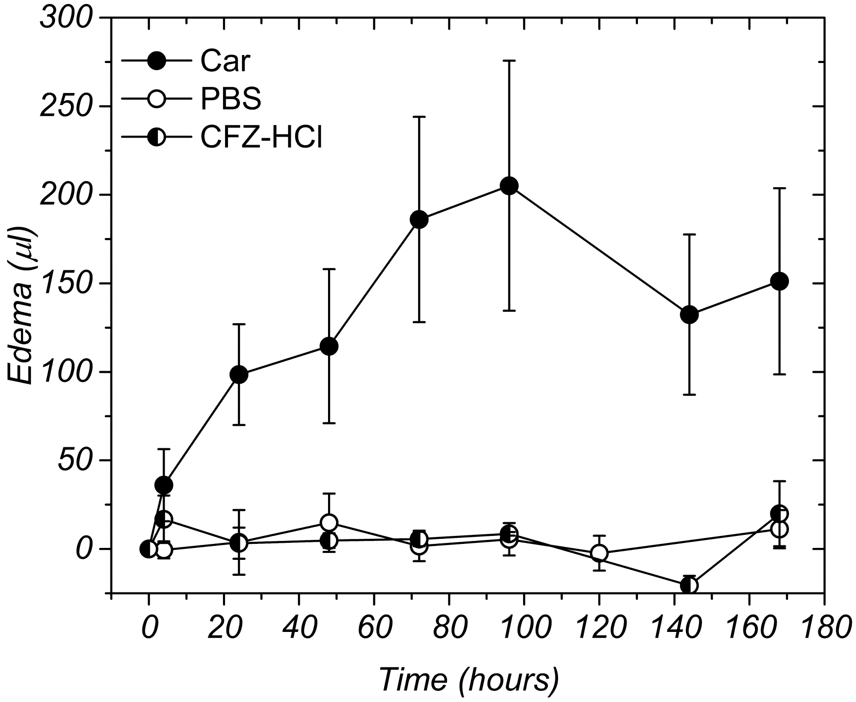

Supplementary Figure S8. CFZ-HCl injections alone. Mice (n=3) were injected with carrageenan (Car, 2% in PBS, 30 μl) or CFZ-HCl microcrystals (2 mM in PBS, 30 μl) in the right footpad and with PBS (30 μl) in the left footpad at time t=0 h and the swollen volume (edema) was calculated as shown in **Supplementary Fig. S5**. CFZ-HCl injected feet showed no signs of swelling (p>0.10 at all time-points vs PBS injected).

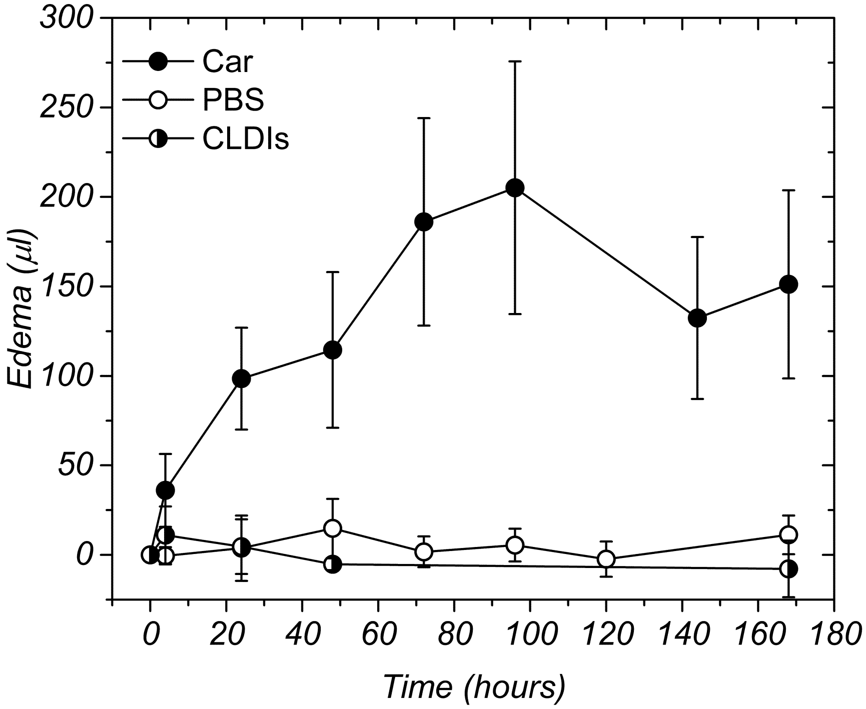

Supplementary Figure S9. CLDI injections alone. Mice (n=3) were injected with carrageenan (Car, 2% in PBS, 30 μl) or CLDI microcrystals (2 mM in PBS, 30 μl) in the right footpad and with PBS (30 μl) in the left footpad at time *t*=0 h and the swollen volume (edema) was calculated as shown in **Supplementary Fig. S5**. CLDI injected feet showed no signs of swelling (all p>0.10 vs PBS injected).

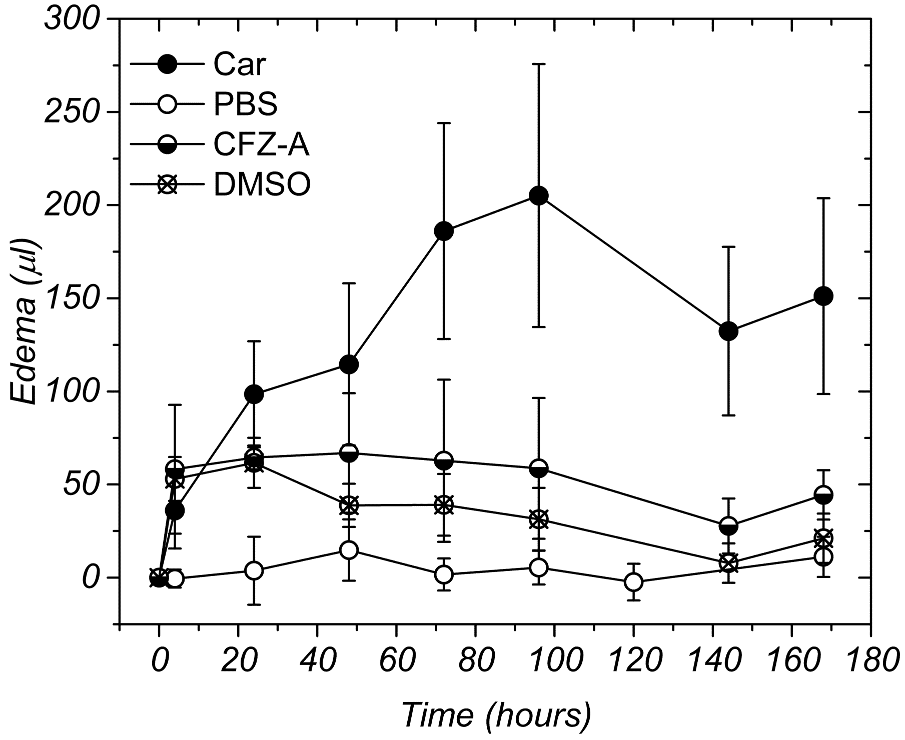

Supplementary Figure S10. CFZ-A and DMSO injections alone. Mice (n=3) were injected with carrageenan (Car, 2% in PBS, 30 µl) or CFZ-A (2 mM in DMSO, 30 µl) in the right footpad and with PBS (30 µl) or DMSO (30 µl) in the left footpad at time t=0 h and the swollen volume (edema) was calculated as shown in **Supplementary Fig. S5**. Both CFZ-A and DMSO injected mice showed some swelling ($p<0.05$, t=24 h vs PBS injected) which was reduced considerably by t=96 h ($p>0.1$ vs PBS injected). Both CFZ-A and DMSO related swelling were equivalent at all time points ($p>0.2$ at all time-points, CFZ-A vs DMSO).

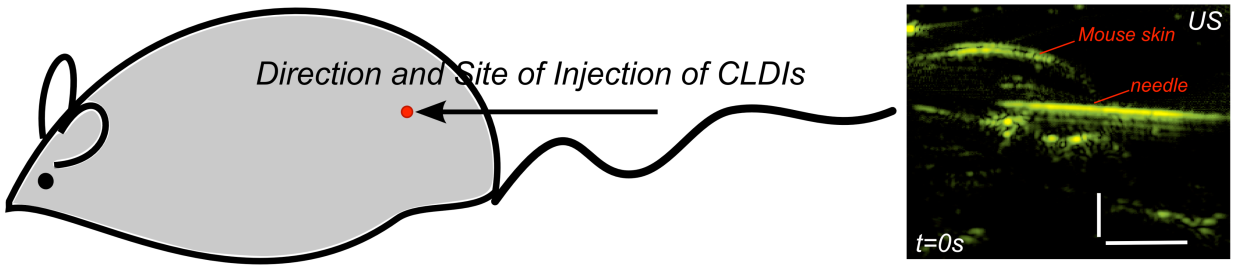

Supplementary Figure S11. Schematic of Mouse Injection and US Image. The mouse was injected with CLDIs in the posterior section of the peritoneal cavity, in close proximity to the hind limbs. Image taken at t=0 s just prior to injection of CFZ-A via US imaging modality is shown alongside. The US image clearly shows the outline of the skin and the needle being inserted (vertical scale – 2 mm, horizontal scale – 5 mm).

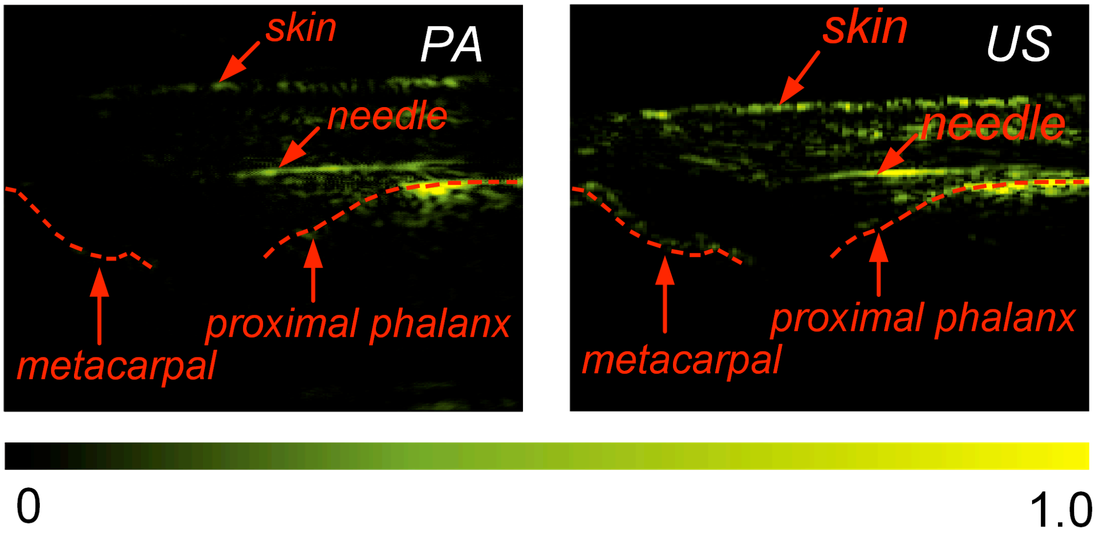

Supplementary Figure S12. Background PA and US Images. Image taken at t=0 s just prior to injection of CFZ-A via both PA (*left*) and US (*right*) imaging modality. Both the bones that form the MCP joint – the proximal phalanx and the metacarpal are visible in the US image.
