# Supplementary figures and images for: Repositioning Clofazimine as a Macrophage-Targeting Photoacoustic Contrast Agent
Source: Sci Rep. 2016 Mar 22;6:23528. doi: 10.1038/srep23528 (PMC4802322; doi:10.1038/srep23528)

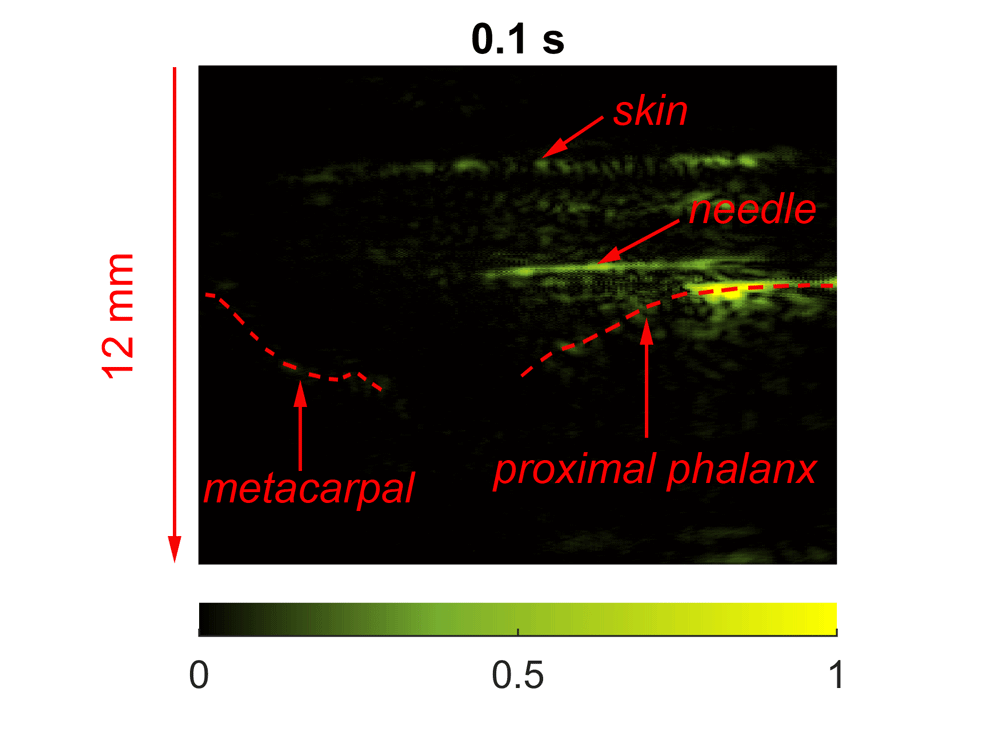

Supplement: Supplementary Movie 1 [file srep23528-s3.gif]
